# Estimation of Overall Survival with Subsequent Treatment Effect by Applying Inverse Probability of Censoring Weighting in the LATITUDE Study

**DOI:** 10.1016/j.euros.2021.11.012

**Published:** 2022-01-06

**Authors:** Yosuke Koroki, Masataka Taguri, Nobuaki Matsubara, Karim Fizazi

**Affiliations:** a Medical Affairs, Janssen Pharmaceutical K.K., Tokyo, Japan; b Graduate School of Data Science, Yokohama City University, Kanagawa, Japan; c Department of Medical Oncology, National Cancer Center Hospital East, Chiba, Japan; d Department of Cancer Medicine, Institut Gustave Roussy, University of Paris Saclay, Villejuif, France

**Keywords:** Abiraterone, Inverse probability of censoring weighting, Metastatic castration-sensitive prostate cancer, Overall survival, Subsequent therapy

## Abstract

**Background:**

In the LATITUDE study (ClinicalTrials.gov, NCT01715285), compared with placebos, abiraterone acetate plus prednisone (AAP) with androgen deprivation therapy (ADT) provided significant overall survival (OS) benefit in high-risk metastatic castration-sensitive prostate cancer (mCSPC) patients. It is controversial whether survival benefits would remain if all patients in the placebo group subsequently received life-extending therapies.

**Objective:**

To estimate treatment effect in the case of all patients in the placebo group receiving life-extending subsequent therapies.

**Design, setting, and participants:**

A post hoc analysis of LATITUDE final-analysis data was carried out (setting and participants have been reported previously).

**Intervention:**

AAP or placebos plus ADT.

**Outcome measurements and statistical analysis:**

We applied the inverse probability of censoring weighting (IPCW) method to represent the situation in which all patients in the placebo group would have received life-extending subsequent therapies. The OS hazard ratio (HR) of AAP versus placebos and associated 95% confidence interval (CI) were estimated using a Cox proportional hazards model.

**Results and limitations:**

Of the 581 eligible patients in the placebo group, 237 (40.8%) did not receive life-extending subsequent therapies. From the unadjusted intention-to-treat analysis, the HR for OS for AAP versus placebos was 0.661 (95% CI 0.564–0.775). Using IPCW to adjust for patients in the placebo group without life-extending subsequent therapies, the HR was 0.732 (95% CI 0.604–0.887). A limitation is a lack of proof that the Cox proportional hazards model for the absence of life-extending subsequent therapy is correctly specified for the IPCW method.

**Conclusions:**

Treatment with AAP exerts OS benefit over placebos in high-risk mCSPC patients, regardless of whether life-extending subsequent therapy is given.

**Patient summary:**

In a previous study, high-risk metastatic castration-sensitive prostate cancer patients who received abiraterone acetate plus prednisone (AAP) with androgen deprivation therapy generally survived longer than those given placebos. The benefit of adding AAP continues regardless of whether life-extending subsequent therapy is given.

## Take Home Messages

An analysis using the inverse probability of censoring weighting showed that the benefits of adding abiraterone acetate plus prednisone to androgen deprivation therapy would have continued even if all patients had received life-extending subsequent therapies for high-risk metastatic castration-sensitive prostate cancer.

## 1 Introduction

Prostate cancer is the second commonest cancer in men worldwide (14.1% of all cancers diagnosed in 2020) and the fifth leading cause of death [1]. Metastatic prostate cancer has a poor prognosis (5-yr survival rate <30%) [2]. Metastatic prostate cancer can be castration-sensitive or castration-resistant (mCSPC and mCRPC, respectively). Androgen deprivation therapy (ADT) is the standard treatment for newly diagnosed, metastatic prostate cancer. However, when first-line ADT fails in mCSPC patients, they are then said to have mCRPC. All mCSPC patients eventually develop mCRPC. Thus, the treatment strategy for mCSPC is clinically important for delaying the development of mCRPC.

New treatments for mCSPC patients are docetaxel [3], [4], abiraterone acetate plus prednisone (AAP) [5], [6], apalutamide [7], and enzalutamide [8], [9] plus ADT, which shows evidence of significant survival benefits. Additionally, docetaxel [10], AAP [11], and enzalutamide [12] plus ADT are available in clinical practice for the treatment of patients with mCRPC, based on evidence of significant survival benefits. When considering survival benefits in mCSPC patients, it is necessary to consider the influence of treatment on outcomes for mCRPC patients.

LATITUDE was the first phase 3 study to examine the survival benefit of adding AAP to ADT in patients with newly diagnosed, high-risk mCSPC. Subsequent therapy for mCRPC was permitted at the investigator’s discretion, after study treatment discontinuation.

Overall survival (OS) at final analysis was greater in the AAP group than in the placebo group: hazard ratio (HR) 0.66 (95% confidence interval [CI] 0.56–0.78; *p* < 0.0001) [5]. Although 57% of patients in the placebo group received at least one life-extending subsequent therapy [5], there remained about 40% of patients in the placebo group who did not receive subsequent therapy. The low proportion of patients who received life-extending subsequent therapy raised the question of whether survival benefits would have remained if all patients had received life-extending subsequent therapy [13].

It is notable that such a large minority of patients in the placebo group did not receive subsequent therapy, despite disease progression with ADT alone (ie, mCRPC) and the availability of various options for the treatment of mCRPC. We consider this issue, originally raised in response to publication of the results of the LATITUDE study [13], to be relevant. Therefore, we conducted the present study, using the inverse probability of censoring weighting (IPCW) method, to clarify how OS would have been affected if all patients in the placebo group had received life-extending subsequent therapy for mCRPC.

In clinical studies, sensitivity analyses for OS are usually conducted to estimate true treatment effect. IPCW is used to deal with informative censoring bias [14]. In oncology clinical studies, it represents the situation where crossover does not occur from control treatments to experimental treatments in all patients. Therefore, IPCW is employed to compare the true treatment effect between experimental and control groups without a crossover bias [15]. Results of the post hoc analysis of the LATITUDE study, adjusting for crossover using IPCW, have confirmed a survival benefit of adding AAP to ADT in high-risk mCSPC patients (HR 0.616; 95% CI 0.524–0.724) [16]. Such a result is inevitable when survival benefits are demonstrated in the unadjusted intention-to-treat (ITT) population.

We applied IPCW to adjust for patients in the placebo group who did not receive life-extending subsequent therapies and estimated OS after considering these subsequent treatment effects, representing the situation where all patients in the placebo group would have received life-extending subsequent therapies.

## 2 Patients and methods

### 2.1 Study design

LATITUDE (ClinicalTrials.gov, NCT01715285) is a double-blind, placebo-controlled phase 3 study in which 1199 patients were randomly assigned to either abiraterone acetate (1000 mg once daily orally) plus prednisone (5 mg once daily orally) plus ADT (AAP group) or dual placebos plus ADT (placebo group; for details see [5]). The AAP and placebo groups included 597 and 602 patients, respectively. The coprimary endpoints were OS and radiographic progression-free survival. The final-analysis datasets were used for the present study.

### 2.2 Statistical analysis

Statistical analyses were performed using SAS version 9.4 (SAS Institute, Cary, NC, USA) in the ITT population (ie, all randomized patients). The primary endpoint in this post hoc analysis was OS for the AAP group (unadjusted) versus the placebo group (adjusted by IPCW). To understand the effect of IPCW, OS for the AAP group (unadjusted) versus the placebo group (adjusted by naïve censoring) was determined. In addition, OS for the AAP group versus the placebo group (when IPCW vs naïve censoring was used) was determined. OS was defined as the time from randomization to death from any cause. For live patients, those lost to follow-up, or those who withdrew consent up to the time of analysis, the last date the patient was known to be alive was censored. Survival distribution and median OS were estimated by the Kaplan-Meier analysis. HRs and 95% CIs were estimated using a stratified Cox proportional hazards model, according to LATITUDE stratification factors [5].

Life-extending subsequent therapies included AAP, enzalutamide, docetaxel, cabazitaxel, and radium-223. In a naïve censoring approach, patients who did not receive life-extending subsequent therapies were censored at the discontinuation of study treatment because we wanted to estimate the survival curves under conditions in which all patients would have received life-extending subsequent therapies unless they died possibly for reasons related to study treatment discontinuation.

In an IPCW analysis, patients who remained after applying naïve censoring were weighted to compensate for missing data. The bias introduced by this informative censoring was corrected by weighting each patient by the inverse of his predicted probability of not being censored at a given time. To adjust for the potential bias from informative censoring, time-dependent stabilized weights were estimated using a Cox proportional hazards model to assess the probability of not receiving life-extending subsequent therapy at any time interval. The denominator of the stabilized weights was obtained by including baseline and time-varying covariates in the model, whereas only baseline covariates were included for the numerator [17]. Baseline covariates were age, Eastern Cooperative Oncology Group performance status (ECOG PS), Gleason score (GS), region, visceral metastases, prostate-specific antigen, hemoglobin, lactate dehydrogenase (LDH), alanine aminotransferase, aspartate aminotransferase, and Functional Assessment of Cancer Therapy-Prostate. Time-varying covariates were number of bone metastases, symptomatic skeletal event (SSE), total bilirubin, potassium, Brief Pain Inventory-short form (BPI-SF), and Brief Fatigue Inventory (BFI). These weights were then used in a weighted Cox proportional hazards model to estimate the HR and 95% CI of the AAP group versus the placebo group.

## 3 Results

### 3.1 Patient disposition and characteristics

**Fig. 1:**
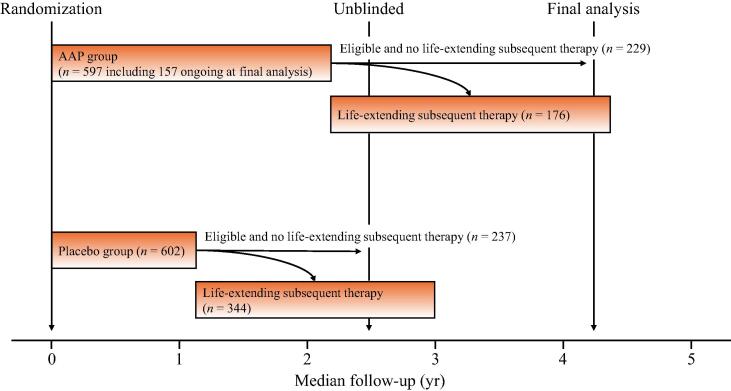
Study design. In the LATITUDE study, patients were randomly assigned to either abiraterone acetate plus prednisone (AAP) plus androgen deprivation therapy (ADT) (the AAP group) or dual placebos plus ADT (the placebo group).

Altogether, respectively, 597 and 602 patients were assigned to the AAP and placebo groups, with 405 and 581 being eligible for life-extending subsequent therapy. Of them, 56.5% (229/405) and 40.8% (237/581), respectively, did not receive such therapies (Fig. 1). Patient characteristics are shown in Table 1. Among patients who discontinued study treatment, those with ECOG PS of 2 and those from Eastern Europe had a greater proportion that did not receive life-extending subsequent therapy. The opposite tendency was seen in those with GS above 8 and from Western Europe in both the AAP and the placebo group. In the two groups, the other patient characteristics were well balanced between patients with and without life-extending subsequent therapy.

**Table 1:** Patient characteristics^a^

Characteristic	AAP group (*n* = 597)			Placebo group (*n* = 602)	
	Ongoing (*n* = 157)	Discontinuation without death (*n* = 405)		Discontinuation without death (*n* = 581)	
		Subsequent therapy (*n* = 176)	Eligible and no subsequent therapy (*n* = 229)	Subsequent therapy (*n* = 344)	Eligible and no subsequent therapy (*n* = 237)
Age (yr), median (range)	66.0 (46–81)	66.0 (44–86)	71.0 (38–89)	66.0 (36–88)	68.0 (33–92)
ECOG PS at baseline, *n* (%)					
0 or 1	153 (97.5)	172 (97.7)	218 (95.2)	340 (98.8)	226 (95.4)
2	4 (2.5)	4 (2.3)	11 (4.8)	4 (1.2)	11 (4.6)
GS at initial diagnosis, *n* (%)					
<8	5 (3.2)	2 (1.1)	6 (2.6)	8 (2.3)	7 (3.0)
8	80 (51.0)	63 (35.8)	107 (46.7)	148 (43.0)	121 (51.1)
>8	72 (45.9)	111 (63.1)	116 (50.7)	188 (54.7)	109 (46.0)
Bone lesions at baseline, *n* (%)					
≤10	78 (49.7)	47 (26.7)	75 (32.8)	130 (37.8)	83 (35.0)
>10	79 (50.3)	129 (73.3)	154 (67.2)	214 (62.2)	154 (65.0)
Presence of visceral disease, *n* (%)					
Yes	27 (17.2)	31 (17.6)	46 (20.1)	62 (18.0)	47 (19.8)
No	130 (82.8)	145 (82.4)	183 (79.9)	282 (82.0)	190 (80.2)
Extent of disease at initial diagnosis, *n* (%)					
Liver	5 (3.2)	10 (5.7)	14 (6.1)	12 (3.5)	15 (6.3)
Lung	20 (12.7)	11 (6.3)	32 (14.0)	40 (11.6)	31 (13.1)
Soft tissue	3 (1.9)	3 (1.7)	2 (0.9)	11 (3.2)	3 (1.3)
Viscera	4 (2.5)	5 (2.8)	9 (3.9)	4 (1.2)	8 (3.4)
Other	0 (0.0)	0 (0.0)	2 (0.9)	0 (0.0)	0 (0.0)
Baseline PSA (ng/ml), median (range)	22.8 (0.04–8775.89)	28.4 (0.12–5381.91)	25.4 (0.14–3732.07)	22.9 (0.05– 8889.60)	24.8 (0.10–4540.10)
Baseline hemoglobin (g/l), median (range)	139.0 (93–172)	131.0 (90–162)	130.0 (90–166)	136.0 (89–172)	130.0 (90–171)
Baseline LDH (U/l), median (range)	173.0 (110–325)	179.0 (73–785)	182.0 (103–1492)	176.0 (67–1349)	177.0 (106–1444)
Baseline ALT (U/l), median (range)	20.0 (6–84)	21.0 (6–85)	18.0 (5–77)	20.0 (4–136)	20.0 (4–96)
Baseline AST (U/l), median (range)	22.0 (11–73)	22.5 (10–58)	22.0 (7–70)	22.5 (8–84)	22.0 (10–74)
Baseline total bilirubin (μmol/l), median (range)	9.0 (3–21)	8.0 (3–29)	8.0 (3–22)	8.0 (3–28)	7.5 (3–29)
Baseline potassium (mmol/l), median (range)	4.4 (3.5–5.9)	4.5 (3.5–5.9)	4.4 (3.5–5.8)	4.4 (3.5–5.8)	4.4 (3.5–5.9)
Total FACT-P score at baseline, mean (SD)	114.4 (18.6)	113.1 (19.9)	109.8 (19.6)	113.1 (18.2)	107.4 (21.6)
Pain score at baseline^b^, mean (SD)	2.0 (2.4)	2.1 (2.2)	2.2 (2.5)	2.1 (2.3)	2.3 (2.5)
Fatigue score at baseline, mean (SD)	1.8 (2.4)	2.3 (2.5)	2.2 (2.6)	2.1 (2.5)	2.3 (2.6)
Region, *n* (%)					
Asia	39 (24.8)	34 (19.3)	46 (20.1)	74 (21.5)	46 (19.4)
Eastern Europe	60 (38.2)	43 (24.4)	97 (42.4)	84 (24.4)	122 (51.5)
Western Europe	34 (21.7)	27 (15.3)	33 (14.4)	61 (17.7)	34 (14.3)
Rest of world	24 (15.3)	72 (40.9)	53 (23.1)	125 (36.3)	35 (14.8)

ALT = alanine aminotransferase; AST = aspartate aminotransferase; ECOG PS = Eastern Cooperative Oncology Group performance status; FACT-P = Functional Assessment of Cancer Therapy-Prostate; GS = Gleason score; LDH = lactate dehydrogenase; PSA = prostate-specific antigen; SD = standard deviation.

a Patients in the abiraterone acetate plus prednisone (AAP) group received AAP plus androgen deprivation therapy (ADT), and patients in the placebo group received dual placebos plus ADT.

b Two patients in each study arm with missing values at the first interim analysis had updated values at the second interim analysis.

The median study treatment durations in the AAP group were 19.9 (range 0.7–61.9) mo in patients with life-extending subsequent therapy and 16.6 (range 0.4–64.2) mo in those without, and those in the placebo group were 15.1 (range 0.9–51.3) mo and 11.6 (range 0.7–49.7) mo, respectively.

### 3.2 Life-extending subsequent therapy

As shown in Table 2, 43.5% (176/405) of patients in the AAP group and 59.2% (344/581) in the placebo group received life-extending subsequent therapy for prostate cancer. Proportions of patients receiving life-extending subsequent therapy tended to be higher in the placebo group than in the AAP group.

**Table 2:** Subsequent therapy for prostate cancer^a^

Variable	AAP group (*n* = 597)		Placebo group (*n* = 602)	
	Subsequent therapy (*n* = 176)	Eligible and no subsequent therapy (*n* = 229)	Subsequent therapy (*n* = 344)	Eligible and no subsequent therapy (*n* = 237)
Total no. of patients with any subsequent therapy, *n* (%)	176 (100.0)	69 (30.1)	344 (100.0)	82 (34.6)
Total no. of patients with systemic subsequent therapy, *n* (%)	176 (100.0)	42 (18.3)	344 (100.0)	56 (23.6)
Life-extending subsequent therapy, *n* (%)	176 (100.0)	0 (0.0)	344 (100.0)	0 (0.0)
Docetaxel	144 (81.8)	0 (0.0)	212 (61.6)	0 (0.0)
Enzalutamide	57 (32.4)	0 (0.0)	99 (28.8)	0 (0.0)
Radium 223 dichloride	27 (15.3)	0 (0.0)	44 (12.8)	0 (0.0)
Cabazitaxel	25 (14.2)	0 (0.0)	50 (14.5)	0 (0.0)
Abiraterone	18 (10.2)	0 (0.0)	156 (45.3)	0 (0.0)
Other subsequent therapy, *n* (%)	71 (40.3)	42 (18.3)	125 (36.3)	56 (23.6)
Bicalutamide	27 (15.3)	30 (13.1)	58 (16.9)	39 (16.5)
Flutamide	3 (1.7)	2 (0.9)	13 (3.8)	8 (3.4)
Other hormonal therapy	6 (3.4)	4 (1.7)	16 (4.7)	6 (2.5)
Other chemotherapy	18 (10.2)	5 (2.2)	30 (8.7)	5 (2.1)
Glucocorticoids	27 (15.3)	3 (1.3)	48 (14.0)	1 (0.4)
Drugs for treatment of bone diseases	9 (5.1)	4 (1.7)	9 (2.6)	2 (0.8)
Investigational drug	7 (4.0)	0 (0.0)	9 (2.6)	0 (0.0)
Others	0 (0.0)	3 (1.3)	4 (1.2)	2 (0.8)
Total no. of patients with subsequent surgery or procedures, *n* (%)	72 (40.9)	36 (15.7)	105 (30.5)	37 (15.6)
Radiotherapy (to bone)	64 (36.4)	30 (13.1)	93 (27.0)	29 (12.2)
Radiotherapy (other than bone)	7 (4.0)	4 (1.7)	13 (3.8)	5 (2.1)
Surgery (to bone)	3 (1.7)	4 (1.7)	4 (1.2)	2 (0.8)
Surgery (other than bone)	2 (1.1)	4 (1.7)	8 (2.3)	4 (1.7)

a Patients in the abiraterone acetate plus prednisone (AAP) group received AAP plus androgen deprivation therapy (ADT), and patients in the placebo group received dual placebos plus ADT.

### 3.3 OS adjusted by IPCW (placebo group only)

**Fig. 2:**
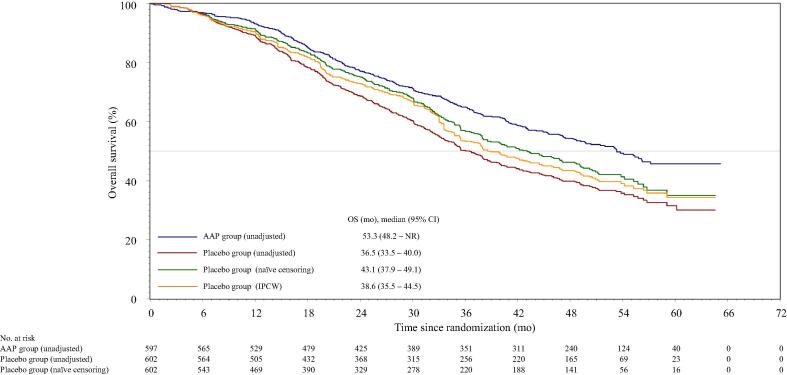
Overall survival (OS) Kaplan-Meier estimates both unadjusted (intention-to-treat analysis) and adjusted for patients in the placebo group who did not receive life-extending subsequent therapy, applying the naïve censoring method and the inverse probability of censoring weighting (IPCW) method. Patients at risk are presented for the naïvely censored curve. Patients at risk are not included for the IPCW curve due to the lack of a clear clinical interpretation of the number of patients at risk associated with the weighted methodology. Patients in the abiraterone acetate plus prednisone (AAP) group received AAP plus androgen deprivation therapy (ADT), and patients in the placebo group received dual placebos plus ADT. CI = confidence interval; NR = not reached.

The final OS in a preplanned ITT analysis was published previously [5]. In the IPCW analysis, the median OS of the placebo group was 38.6 mo (95% CI 35.5–44.5 mo; Fig. 2).

**Fig. 3:**
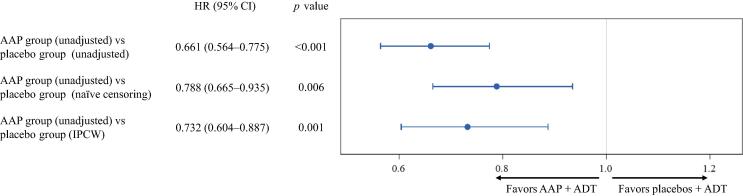
Forest plots both unadjusted and adjusted for patients in the placebo group not receiving life-extending subsequent therapy, applying either naïve censoring or inverse probability of censoring weighting (IPCW). All *p* values are from the hazard ratio (HR) of the Cox proportional hazards model. Patients in the abiraterone acetate plus prednisone (AAP) group received AAP plus androgen deprivation therapy (ADT), and patients in the placebo group received dual placebos plus ADT. CI = confidence interval.

The HR for the AAP group (unadjusted) versus the placebo group (IPCW-adjusted) was 0.732 (95% CI 0.604–0.887), with a nominal *p* value of 0.001 (Fig. 3).

In the naïve censoring analysis, the median OS of the placebo group was 43.1 mo (95% CI 37.9–49.1 mo; Fig. 2). HR for the unadjusted AAP group versus the naïve censoring-adjusted placebo group was 0.788 (95% CI 0.665–0.935), with a nominal *p* value of 0.006 (Fig. 3).

The IPCW method provided a smaller estimate of treatment effect than the unadjusted ITT estimate and a larger estimate of treatment effect than the naïve censoring estimate (Fig. 3).

### 3.4 OS adjusted by IPCW in both groups

In the IPCW analysis, the median OS of the AAP group was not reached (95% CI 53.3 mo–not reached; Supplementary Fig. 1). The HR for the AAP group versus the placebo group (IPCW used for both groups) was 0.643 (95% CI 0.522–0.793; nominal *p* < 0.001; Supplementary Fig. 2).

In the naïve censoring analysis, the median OS of the AAP group was not reached (95% CI 53.7 mo–not reached; Supplementary Fig. 1). The HR for the AAP group versus the placebo group (naïve censoring used for both groups) was 0.658 (95% CI 0.548–0.790; nominal *p* < 0.001; Supplementary Fig. 2).

### 3.5 Probability of the absence of life-extending subsequent therapy for IPCW

Results of the Cox proportional hazards model for the probability of life-extending subsequent therapy based on the IPCW method are shown in Supplementary Table 1. BPI-SF as a time-varying covariate significantly predicted the absence of life-extending subsequent therapy in both the AAP and the placebo group. The SSE and BFI as time-varying covariates were significant predictors of the absence of life-extending subsequent therapy in the placebo group (Supplementary Table 1), whereas age, region (Asia vs Eastern Europe), and LDH as baseline covariates were significant predictors of the absence of life-extending subsequent therapy in the AAP group (Supplementary Table 1).

Estimated stabilized weights for the IPCW method are shown in Supplementary Figure 3. Weights in the AAP group (Supplementary Fig. 3A) tended closer to 1, which means 0 in log weights, than those in the placebo group (Supplementary Fig. 3B).

## 4 Discussion

We estimated the treatment effect of all patients in the placebo group receiving life-extending subsequent therapies. Of the 581 eligible patients in the placebo group, 237 (40.8%), did not receive life-extending subsequent therapies. In the unadjusted ITT analysis, HR for OS for the AAP group versus the placebo group was 0.661 (95% CI 0.564–0.775). HR for the unadjusted AAP group versus the placebo group adjusted by IPCW was 0.732 (95% CI 0.604–0.887).

To our knowledge, this is the first study to use IPCW to adjust for patients who did not receive subsequent therapy and estimate OS while accounting for subsequent treatment effects. This is because IPCW and naïve censoring are usually used to adjust for patients who crossed over, estimating OS in the absence of a crossover treatment effect [14]. Our results showed a consistent treatment benefit from AAP use in high-risk mCSPC patients even if all patients in the placebo group had received life-extending subsequent therapy for mCRPC. This finding is potentially useful when considering AAP use to treat mCSPC rather than mCRPC in clinical practice, because it addresses a critical concern raised after the publication of the results of the LATITUDE study [13]; briefly, our results show that AAP has a treatment benefit in situations closer to those encountered in real-world settings.

Various options are available for the treatment of mCRPC after ADT, and in the real world, about 80% of patients receive life-extending therapy. Although it is unclear why a much smaller proportion (<60%) of patients in the placebo group received subsequent therapy in the LATITUDE study, the results of the present analysis suggest that even if all patients in the placebo group had received subsequent therapy, the OS benefit of AAP treatment for mCSPC over placebo would have remained unchanged. We believe that the present study is meaningful because we have succeeded in confirming, using a causal inference method, the clinical usefulness of AAP treatment for mCSPC in conditions close to those of the real world.

Globally, docetaxel, AAP, enzalutamide, cabazitaxel, and radium-223 are approved for mCRPC treatment, and these life-extending therapies are recommended for mCRPC patients as the highest -level treatment option in guidelines for prostate cancer [18], [19]. Based on these guidelines, 77% (1980/2559) of mCRPC patients received life-extending therapies, of whom 65% were on AAP or enzalutamide as first-line therapy in real-world settings [20]. Thus, since few patients with mCRPC are untreated in the real world, we performed an IPCW analysis to adjust for the effect of the absence of life-extending subsequent therapy in LATITUDE and estimate AAP treatment effect while accounting for life-extending subsequent therapy.

When both groups were adjusted for the effect of the absence of life-extending subsequent therapies, HR was smaller than in the unadjusted ITT analysis (HR 0.643 vs 0.661). However, when just the placebo group was adjusted for the effect of the absence of life-extending subsequent therapies, HR was larger than in the unadjusted ITT analysis (HR 0.732 vs 0.661). These differences in HR were considered to be due to a smaller proportion of life-extending subsequent therapies in the AAP group than in the placebo group (43.5% vs 59.2%).

When the placebo group was unadjusted, the estimated OS incorporated the fact that not all patients received life-extending subsequent therapy. When the placebo group was adjusted by using IPCW, OS was estimated considering that all the patients in the placebo group had received life-extending subsequent therapy. In this case, as subsequent therapy for patients in the placebo group is synonymous with first-line treatment for mCRPC, the difference of 2.1 mo in median OS with and without IPCW adjustment (ie, median OS 36.5 [unadjusted] vs 38.6 [IPCW] mo) signifies the effect that life-extending subsequent therapy would have on the OS of mCRPC patients. This contention is supported by the fact that differences in median OS between the investigational drug and placebo groups were 2–4 mo in clinical studies of mCRPC patients [10], [11], [12], [21], [22], [23], [24].

Moreover, OS results from the IPCW analysis were longer than those from the unadjusted ITT analysis and shorter than those from the naïve censoring analysis, with a significant difference in HR between the AAP and placebo groups, suggesting a bias where life-extending subsequent therapies were not given to patients with a poor prognosis in the placebo group. Such a result would be plausible in considering the median study treatment duration (15.1 mo with life-extending subsequent therapy vs 11.6 mo in the absence of life-extending subsequent therapy) and the estimates of the Cox proportional hazards model for IPCW (HR for SSE 4.647, HR for BPI-SF 1.137, and HR for BFI 1.181). The IPCW analysis, which accounted for selective crossover, may have adjusted for bias and detected an OS benefit that otherwise would not have been detected with an unadjusted ITT or naïve censoring analysis [15].

Rank-preserving structural failure time (RPSFT) models [25] and the iterative parameter estimation (IPE) algorithm [26] have been used to adjust for crossover treatment effects, with a potential confounding effect on a survival analysis. However, these analyses are required to comply with common treatment effects: the assumption that the treatment effect of a drug administrated to a patient is the same, regardless of treatment order. Since the treatment effect of AAP is not the same in mCSPC (OS in LATITUDE [5], extra 16.8 mo; AAP vs placebos) and mCRPC (OS before chemotherapy in COU-AA-302 [11], 4.4 mo extra; AAP vs placebo, and OS after chemotherapy in the COU-AA-301 study [21], 4.6 mo extra; AAP vs placebo), the treatment effect of AAP differs significantly with treatment order, indicating that essential assumptions for use of an RPSFT model or IPE analysis were not met in LATITUDE. It was thus considered appropriate to use IPCW to adjust for the absence of subsequent treatment effects in this study.

The use of the IPCW model is considered to be more appropriate in studies with a large sample size and moderate proportion of patients with crossover, when the assumption of no unmeasured confounders is met [27]. Since the assumption of no unmeasured confounders is essential for IPCW analysis [28], both baseline and time-varying covariates were selected based on clinical importance, and the necessary covariates were included in the analysis model for estimating the probability of the absence of life-extending subsequent therapy. However, model specifications were not confirmed. This was a study limitation: the lack of proof that the Cox proportional hazards model for the absence of life-extending subsequent therapy is specified correctly for the IPCW method.

Although the proportion of patients without life-extending subsequent therapies was not small, it was not considered a barrier to use of the IPCW analysis because the sample size of LATITUDE was large and the 95% CI of the HR in the naïve censoring analysis was not substantially different from that in the unadjusted ITT analysis.

In this study, the probability of censoring was estimated using the Cox proportional hazards model. This model, based on multiple explanatory variables, was considered appropriate because of the timing of censoring (ie, without life-extending subsequent therapy) varied between patients [14].

## 5 Conclusions

The addition of AAP to ADT in high-risk mCSPC patients was beneficial, regardless of the receipt of life-extending subsequent therapy. IPCW is useful for estimating OS with subsequent treatment effects in clinical studies where a small population receives subsequent therapy.

  ***Author contributions:*** Yosuke Koroki had full access to all the data in the study and takes responsibility for the integrity of the data and the accuracy of the data analysis.

*Study concept and design:* Koroki, Taguri.

*Acquisition of data:* Matsubara, Fizazi.

*Analysis and interpretation of data:* Koroki, Taguri, Matsubara, Fizazi.

*Drafting of the manuscript:* Koroki.

*Critical revision of the manuscript for important intellectual content:* Koroki, Taguri, Matsubara, Fizazi.

*Statistical analysis:* Koroki.

*Obtaining funding:* Koroki.

*Administrative, technical, or material support:* Koroki.

*Supervision:* Taguri.

*Other:* None.

  ***Financial disclosures:*** Yosuke Koroki certifies that all conflicts of interest, including specific financial interests and relationships and affiliations relevant to the subject matter or materials discussed in the manuscript (eg, employment/affiliation, grants or funding, consultancies, honoraria, stock ownership or options, expert testimony, royalties, or patents filed, received, or pending), are the following: Yosuke Koroki is an employee of Janssen Pharmaceutical K.K. and holds stock in Johnson & Johnson. Masataka Taguri has nothing to disclose. Nobuaki Matsubara has reported advisory roles for Janssen, Sanofi, AstraZeneca, Roche, and Pfizer; speakers’ bureau for Janssen and Sanofi; and research grant/funding (institutional) from Janssen, AstraZeneca, Takeda, Lilly, Amgen, Astellas, Chugai, Bayer, MSD, Ono, Taiho, and Pfizer. Karim Fizazi has reported participation in advisory boards for Amgen, Astellas, AstraZeneca, Advanced Accelerator Applications, Bayer, Clovis, CureVac, ESSA, Genentech, Janssen, MSD, Orion, and Sanofi.

  ***Funding/Support and role of the sponsor*:** The LATITUDE study was funded by Janssen Research & Development. Editorial support was funded by Janssen Pharmaceutical K.K.

  ***Acknowledgments*:** We thank the study participants and their families, as well as the investigators, study coordinators, study teams, and nurses.
