# Supplementary figures and images for: Estimation of Overall Survival with Subsequent Treatment Effect by Applying Inverse Probability of Censoring Weighting in the LATITUDE Study
Source: Eur Urol Open Sci. 2022 Jan 6;36:51–8. doi: 10.1016/j.euros.2021.11.012 (PMC8783036; doi:10.1016/j.euros.2021.11.012)

Supplementary Fig. 1

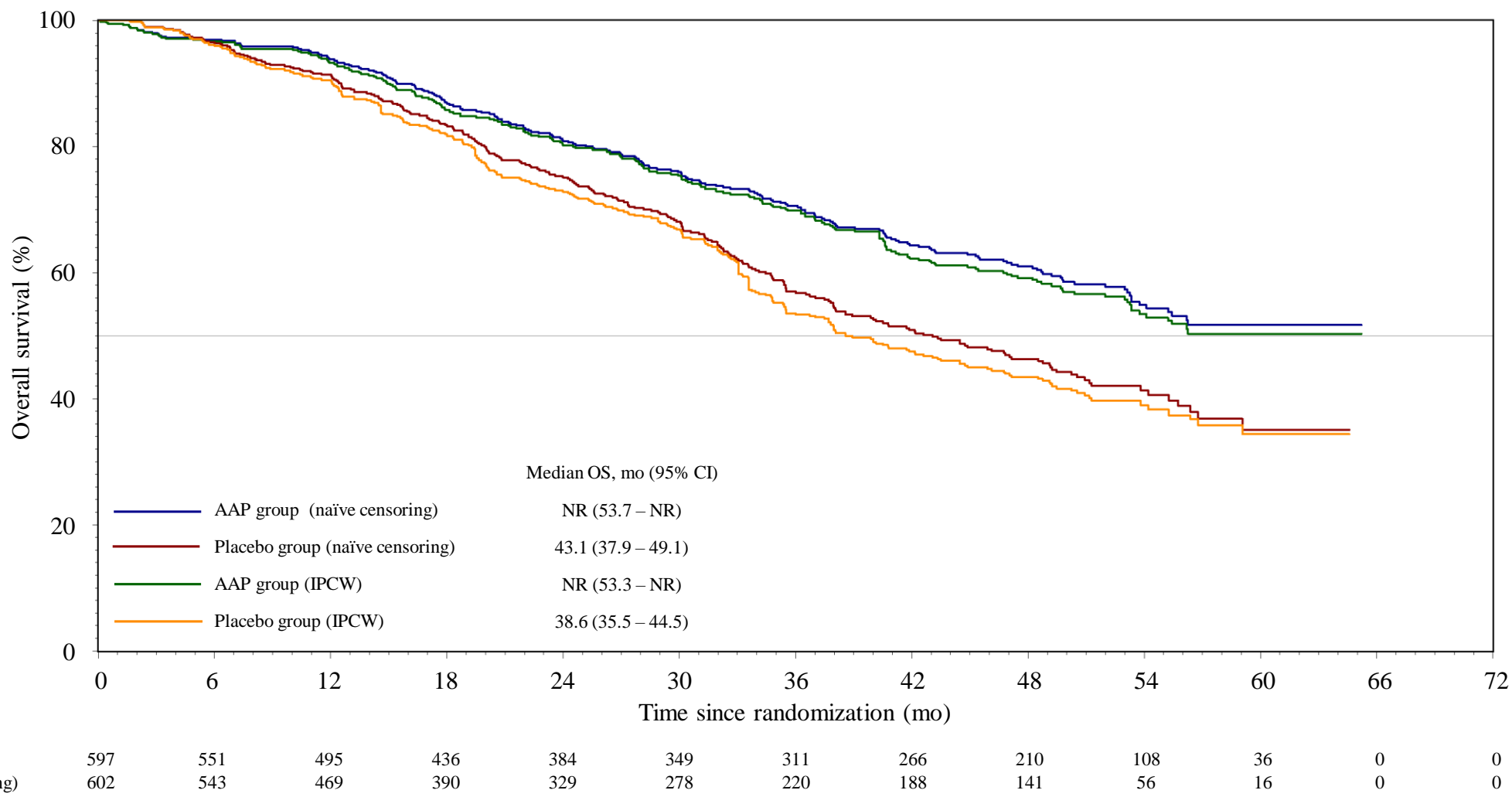

Supplementary Fig. 2

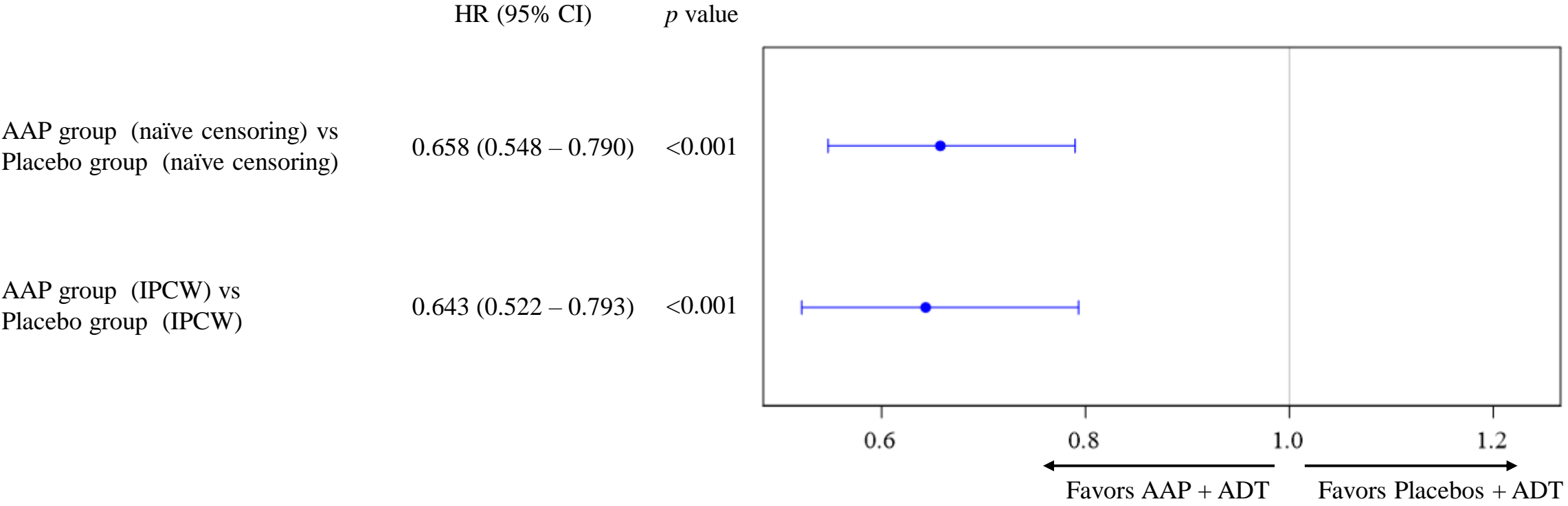

**Supplementary Fig. 3**

**A AAP group**

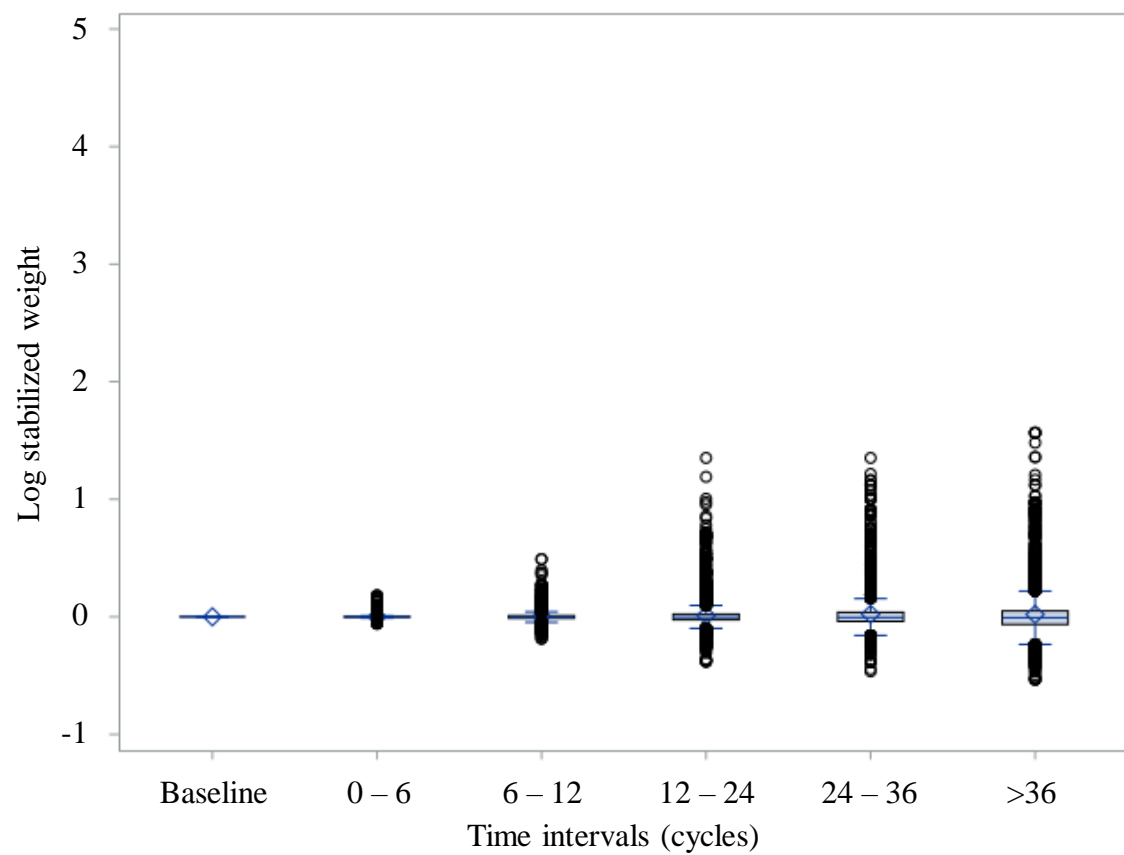

**B Placebo group**

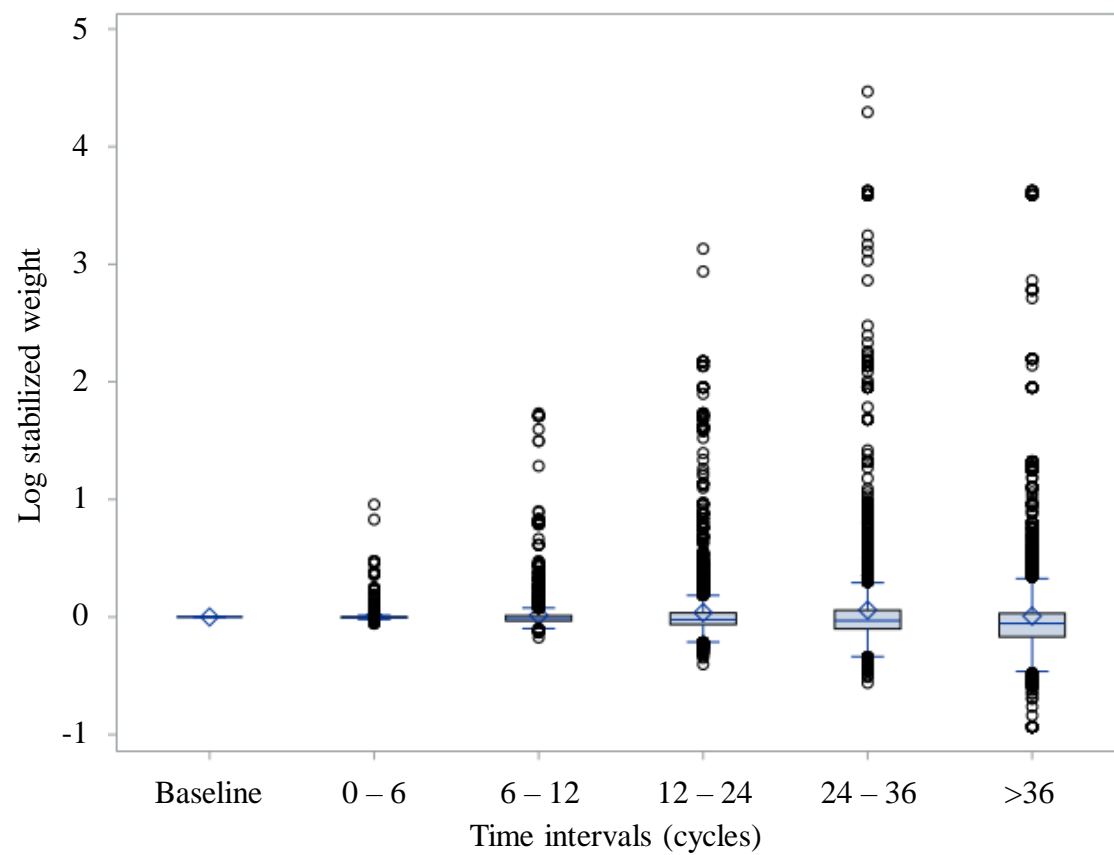

Supplement: Supplementary data 1 [file mmc1.pdf]
