# Supplementary material for: Estimation of Overall Survival with Subsequent Treatment Effect by Applying Inverse Probability of Censoring Weighting in the LATITUDE Study
Source: Eur Urol Open Sci. 2022 Jan 6;36:51–8. doi: 10.1016/j.euros.2021.11.012 (PMC8783036; doi:10.1016/j.euros.2021.11.012)
Supplement: Supplementary data 2 [file mmc2.docx]

**Supplementary Table S1 –** Cox proportional hazards model for the probability of the absence of life-extending subsequent therapy for the inverse probability of censoring weighting method (A) in the AAP group and (B) in the placebo group^a^

A

| AAP group | Model fit | | Hazard ratio | |
| --- | --- | --- | --- | --- |
|  | Coefficient (SE) | *p* value | Estimate | 95% CI |
| Baseline covariates |  |  |  |  |
| Age | 0.056 (0.012) | <0.0001 | 1.058 | 1.034–1.082 |
| ECOG PS (0, 1 vs 2) | 0.475 (0.464) | 0.3053 | 1.609 | 0.648–3.992 |
| GS (8 vs <8) | –0.465 (0.679) | 0.4934 | 0.628 | 0.166–2.378 |
| GS (8 vs >8) | –0.021 (0.198) | 0.9162 | 0.979 | 0.664–1.444 |
| Region (Asia vs Eastern Europe) | 0.757 (0.297) | 0.0109 | 2.133 | 1.191–3.821 |
| Region (Asia vs Western Europe) | 0.605 (0.324) | 0.0615 | 1.831 | 0.971–3.453 |
| Region (Asia vs Rest of world) | 0.517 (0.370) | 0.1619 | 1.677 | 0.813–3.461 |
| Visceral metastases (No vs Yes) | 0.095 (0.259) | 0.7129 | 1.100 | 0.662–1.828 |
| PSA (ng/ml) | –0.000 (0.000) | 0.3299 | 1.000 | 1.000–1.000 |
| Hemoglobin (g/l) | –0.011 (0.006) | 0.0809 | 0.989 | 0.976–1.001 |
| LDH (U/l) | 0.002 (0.001) | 0.0317 | 1.002 | 1.000–1.003 |
| ALT (U/l) | 0.001 (0.012) | 0.9584 | 1.001 | 0.977–1.025 |
| AST (U/l) | –0.015 (0.020) | 0.4479 | 0.985 | 0.948–1.024 |
| FACT-P | 0.004 (0.006) | 0.4950 | 1.004 | 0.992–1.016 |
| Time-varying covariates |  |  |  |  |
| No. of bone metastases | 0.005 (0.016) | 0.7631 | 1.005 | 0.974–1.037 |
| SSE | 0.642 (0.374) | 0.0860 | 1.900 | 0.913–3.954 |
| Total bilirubin (μmol/l) | –0.039 (0.033) | 0.2306 | 0.962 | 0.902–1.025 |
| Potassium (mmol/l) | –0.401 (0.231) | 0.0821 | 0.670 | 0.426–1.052 |
| BPI-SF | 0.141 (0.050) | 0.0044 | 1.151 | 1.045–1.269 |
| BFI | 0.056 (0.052) | 0.2875 | 1.057 | 0.954–1.172 |

B

| Placebo group | Model fit | | Hazard ratio | |
| --- | --- | --- | --- | --- |
|  | Coefficient (SE) | *p* value | Estimate | 95% CI |
| Baseline covariates |  |  |  |  |
| Age | –0.006 (0.011) | 0.6045 | 0.994 | 0.973–1.016 |
| ECOG PS (0, 1 vs 2) | –0.078 (0.532) | 0.8830 | 0.925 | 0.326–2.624 |
| GS (8 vs <8) | –0.182 (0.582) | 0.7550 | 0.834 | 0.266–2.610 |
| GS (8 vs >8) | –0.243 (0.199) | 0.2220 | 0.784 | 0.530–1.159 |
| Region (Asia vs Eastern Europe) | 0.201 (0.242) | 0.4053 | 1.223 | 0.762–1.963 |
| Region (Asia vs Western Europe) | –0.210 (0.300) | 0.4844 | 0.811 | 0.450–1.460 |
| Region (Asia vs Rest of world) | –0.041 (0.301) | 0.8927 | 0.960 | 0.532–1.732 |
| Visceral metastases (No vs Yes) | –0.020 (0.273) | 0.9410 | 0.980 | 0.574–1.674 |
| PSA (ng/ml) | 0.000 (0.000) | 0.5965 | 1.000 | 1.000–1.000 |
| Hemoglobin (g/l) | –0.009 (0.007) | 0.1785 | 0.991 | 0.978–1.004 |
| LDH (U/l) | –0.000 (0.001) | 0.9144 | 1.000 | 0.999–1.001 |
| ALT (U/l) | 0.004 (0.009) | 0.6671 | 1.004 | 0.986–1.022 |
| AST (U/l) | –0.001 (0.014) | 0.9528 | 0.999 | 0.973–1.026 |
| FACT-P | 0.003 (0.006) | 0.5717 | 1.003 | 0.992–1.015 |
| Time-varying covariates |  |  |  |  |
| No. of bone metastases | –0.001 (0.015) | 0.9662 | 0.999 | 0.970–1.030 |
| SSE | 1.536 (0.319) | <0.0001 | 4.647 | 2.486–8.684 |
| Total bilirubin (μmol/l) | 0.010 (0.028) | 0.7130 | 1.010 | 0.957–1.067 |
| Potassium (mmol/l) | 0.010 (0.226) | 0.9658 | 1.010 | 0.649–1.572 |
| BPI-SF | 0.128 (0.042) | 0.0022 | 1.137 | 1.047–1.234 |
| BFI | 0.166 (0.049) | 0.0007 | 1.181 | 1.072–1.301 |

ALT, alanine aminotransferase; AST, aspartate aminotransferase; BFI, Brief Fatigue Inventory; BPI-SF, Brief Pain Inventory short form; CI, confidence interval; ECOG PS, Eastern Cooperative Oncology Group Performance Status; FACT-P, Functional Assessment of Cancer Therapy-Prostate; GS, Gleason score; LDH, lactate dehydrogenase; PSA, prostate-specific antigen; SSE, symptomatic skeletal event.

^a^ Patients in the abiraterone acetate plus prednisone (AAP) group received AAP plus androgen deprivation therapy (ADT), and patients in the placebo group received dual placebos plus ADT. Model-dependent variable is time to the absence of life-extending subsequent therapy, expressed as months from date of randomization to date of discontinuation of study treatment in cases of patients who did not receive life-extending subsequent therapy. If the hazard ratio is <1, then the result favors the first level of the parameter (as listed above).
